# Comprehensive Risk Assessment of Schistosomiasis Epidemic Based on Precise Identification of *Oncomelania hupensis* Breeding Grounds—A Case Study of Dongting Lake Area

**DOI:** 10.3390/ijerph18041950

**Published:** 2021-02-17

**Authors:** Jun Xu, Xiao Ouyang, Qingyun He, Guoen Wei

**Affiliations:** 1 College of Resources and Environmental Sciences, Hunan Normal University, Changsha 410081, China; xujun19900531@gmail.com; 2 Hunan Institute of Economic Geography, Hunan University of Finance and Economics, Changsha 410205, China; 3 College of Geography and Ocean Sciences, Nanjing University, Nanjing 210023, China; dg1927034@smail.nju.edu.cn

**Keywords:** breeding grounds, comprehensive risk of epidemic, spatio-temporal evolution, disease geography, schistosomiasis

## Abstract

Spatio-temporal epidemic simulation, assessment, and risk monitoring serve as the core to establishing and improving the national public health emergency management system. In this study, we investigated *Oncomelania hupensis* breeding grounds and analyzed the locational and environmental preferences of snail breeding in Dongting Lake (DTL), Hunan, China. Using geographic information systems and remote sensing technology, we identified schistosomiasis risk areas and explored the factors affecting the occurrence and transmission of the disease. Several key conclusions were drawn. (1) From 2006 to 2016, the spatial change of potential *O. hupensis* breeding risk showed a diminishing trend from the eastern and northern regions to southwest DTL. Environmental changes in the eastern DTL region resulted in the lakeside and hydrophilic agglomerations of the *O. hupensis* populations. The shift in snail breeding grounds from a fragmented to centralized distribution indicates the weakening mobility of the *O. hupensis* population, the increasing independence of solitary groups, and the growing dependence of the snail population to the local environment. (2) The spatial risk distribution showed a descending gradient from west Dongting area to the east and an overall pattern of high in the periphery of large lakes and low in other areas. The cold-spot areas had their cores in Huarong County and Anxiang County and were scattered throughout the peripheral areas. The hot-spot areas had their center at Jinshi City, Nanxian County, and the southern part of Huarong County. The areas with increased comprehensive risks changed from centralized and large-scale development to fragmented shrinkage with increased partialization in the core area. The risk distribution’s center shifted to the northwest. The spatial risk distribution exhibited enhanced concentricity along the major axis and increased dispersion along the minor axis.

## 1. Introduction

In recent years, researchers from around the world including those in the field of geography have focused their research on health-related topics such as epidemic assessment, risk monitoring, and time–space simulation of regional epidemics, helpful in sound public health emergency management [1,2,3]. One natural focal disease with a long history and wide-ranging effect in many parts of the world is Schistosomiasis Japonica (hereinafter referred to as schistosomiasis). In China, schistosomiasis is mainly distributed in 12 provinces (or autonomous regions and municipalities) of the Yangtze River and the south of the Yangtze River including Jiangsu, Zhejiang, Anhui, Jiangxi, Hubei, Hunan, Sichuan, Guangxi, and Yunnan. This disease is seriously detrimental to people’s health and hinders the socio-economic development of affected areas [4]. Monitoring and identifying risks of an epidemic and reducing its effect on the population’s health and well-being is key to achieving a “Healthy China Strategy” and ensuring high-quality life and human development.

In terms of prevention and cure, the treatment of diseases falls under the realm of medicine, while prevention and control is a systematic regional venture involving multiple disciplines and fields. Since the 20th century, researchers in disease geography and spatial epidemiology have carried out numerous studies on schistosomiasis prevention and control. For instance, De et al. (2012) used QuickBird panchromatic and multispectral images to quantitatively evaluate land-use and, with geographic information systems (GIS), analyzed changes in schistosomiasis-endemic areas [5]. Walz Yvonne and Wegmann (2015) adopted high-resolution remote sensing (RS) data to establish habitat suitability index-based models [6]. They found that RS data could be updated periodically through empirical analysis to monitor potential new hot-spots of schistosomiasis transmission. Wang et al. (2012) used regression analysis combined with geostatistics to quantitatively characterize the spatial information of *O. hupensis* distributions with environmental factor indicators [7]. They established the relationship between spatial environmental factors and snail distribution to establish a risk model and a prediction model of snail distribution. Xia et al. (2017) collected data of *O. hupensis* breeding grounds in the Poyang Lake area and related environmental factors to construct a maximum entropy niche model to generate a distribution map of *O. hupensis* breeding grounds [8].

As an epidemic with pronounced geographic characteristics, schistosomiasis is theoretically suitable for the simulation study of the spatial evolution of epidemic areas. Regarding technology and research, the focus of schistosomiasis control in China has shifted from epidemic data analysis to micro-ecology, epidemiology, and geographic research, and from locally focused management toward global monitoring and prevention [9]. Currently, more focus has been given to county, town, and village-scale epidemic studies, while larger areas such as river and lake basin levels have been overlooked.

The monitoring of *O. hupensis* and its breeding environment has become essential in schistosomiasis prevention and mitigation of “human–land contradiction” between potable and infested water. In recent years, the human–land contradiction between *O. hupensis* breeding grounds and human–animal activities has become the focus of risk control and epidemic rebound management [4,10]. As a natural focal disease, research on response factors for schistosomiasis and its hidden interaction mechanisms can help develop early warning and assessment capabilities and improve major epidemic schistosomiasis prevention and control systems and mechanisms [11,12].

Currently, the schistosomiasis epidemic in the Dongting Lake (DTL) area of Hunan Province has been controlled and mitigated, and its prevention and control have shifted from transmission control to transmission interruption. Plans and strategies are in place toward eliminating schistosomiasis. However, based on the Schistosomiasis Elimination Plan of Hunan Province (2016–2025), schistosomiasis elimination should be reached by 2025, which means that existing schistosomiasis control policy and strategies must be adjusted appropriately [10,13]. Studying the spatio-temporal evolution characteristics of schistosomiasis risk is crucial to achieving schistosomiasis elimination. 

To address this need, this study used the DTL area as the research object and applied spectral feature analysis, data mining and fusion, and spatial analysis in GIS to identify potential epidemic risk areas. We analyzed the snails and their preferred breeding locations using spectral index. Quantitative assessment and schistosomiasis risk simulation in the DTL area from 2006 to 2016 were conducted based on epidemic and susceptibility indexes obtained from epidemiological and land-use data. The results from this study can be used to improve the sensitivity and accuracy of risk identification, particularly in low-level epidemic areas.

## 2. Materials and Methods

### 2.1. Research Framework

Risk detection and quantitative assessment are crucial in monitoring and early warning development in the regional public health emergency management system [14,15,16]. This study analyzed the epidemic source, carrier, and motivation of schistosomiasis in the aspects of epidemiology, geography, and ecology. By defining the *O. hupensis* breeding risk, regional susceptibility risk, and epidemic prevalence risk, and by following the ‘understanding—identification—monitoring—optimization’ strategy, we combined the spectral feature analysis, data mining and fusion, spatial analysis, and other GIS functions to identify and assess the comprehensive risk of schistosomiasis epidemic in the DTL area. We then conducted zoning analysis on spatio-temporal distribution and control strategies for schistosomiasis. 

Schistosomiasis epidemic is a complex systemic problem, which is affected by various risks. The main aspects of risks analyzed in this study were *O. hupensis* breeding risk, epidemic risk, and susceptibility of land-use mainly for the following reasons.

(1) Since *O. hupensis*, patients, livestock, and contaminated water can be potentially exposed to infection and have strong spatiotemporal dynamics, evaluating the complex relationships of these factors is important in understanding the schistosomiasis risks [17,18]. Simply studying a single parameter’s activity patterns cannot accurately reveal the potential risks for an epidemic outbreak. However, the breeding grounds of *O. hupensis* can be monitored as the epidemic source using the environmental detection of important sources for the breeding and spread of *O. hupensis*.
(2) The development and change of an epidemic is a long-term, spatio-temporal, cause-and-effect process and is closely related to snail status, patients, and sick animals [19]. The epidemic risk data, comprehensively defined based on epidemiological data, can be used to measure the influence of various factors including snail distribution, snail density, number of patients, number of sick animals, and their activities.
(3) Epidemic factors are both complex and changeable. Schistosomiasis distribution characteristics, patterns, and trends vary considerably for different regions, prevalence types, and socio-economic attributes [20,21]. Hence, the sensitivity of different types of ground objects to changes in the epidemic was determined. We also measured the susceptibility for the different regions based on the combination of land-use types to quantify the potential risks of epidemic carriers.

**Figure 1:**
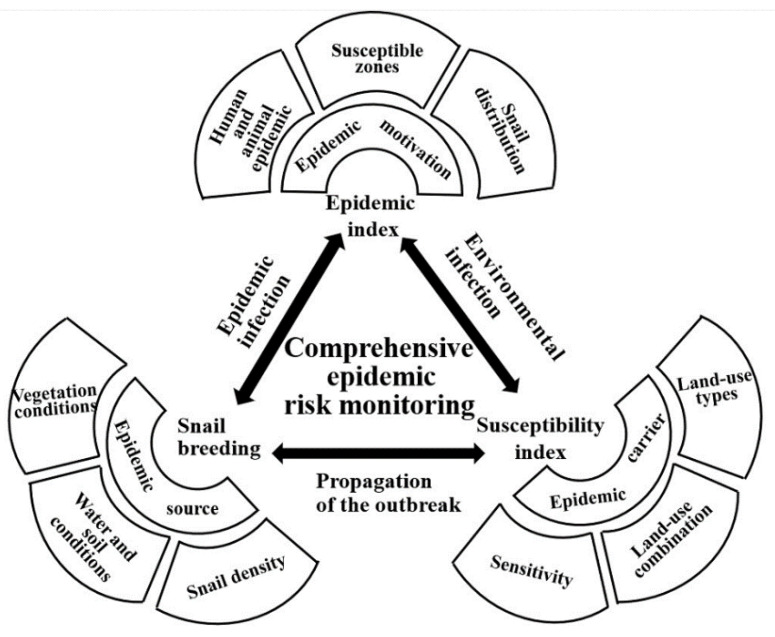
Comprehensive risk formation mechanism and monitoring framework of epidemic area.

Aside from these parameters, the interaction of epidemic risk, susceptibility risk, and snail breeding risk can be used to determine the dynamic changes of schistosomiasis risk in the DTL area. Specifically, the *O. hupensis* breeding environment serves as the source factor responsible for the occurrence, prevalence, and spread of schistosomiasis, which, to a certain extent, determines the probability of a regional schistosomiasis epidemic. Epidemic risk, based on regional historical epidemiological data, and human–land contradiction based on the regional land-use types, can be used to characterize and evaluate the development potential, driving force, and carrier of the epidemic. The superposition of these parameters was used for the comprehensive risk assessment of the schistosomiasis epidemic in the DTL area, as shown in Figure 1.

### 2.2. Research Methods

#### 2.2.1. Construction of the Grid System

This study conducted a quantitative assessment of integrated risks in an epidemic from three dimensions: snail brewing risk, epidemiological risk, and susceptibility based on land use. It involves many kinds of sub-data with varying spatial resolution. Therefore, by constructing a hexagonal grid system, we can unify the quantization scale of multi-source data in the grid system to facilitate the matching and operation of data with different spatial resolutions. It can also ensure fitting for grid systems with irregular edges and maintain spatial balance in the mosaic. The method of grid system is widely used in the fields of ecology, economics, and geography. This paper was based on the related research results published by Xu, Ouyang, and He [22,23,24,25,26,27].

We utilized the hexagons fishnet tool (Tessellation) of ArcGIS 10.2 and established a grid system consisting of 5887 hexagonal cells covering the whole area. Each cell was 2 km wide from east to west, 2.32 km long from north to south, an area of 3.48 km^2^, and a 2-km distance between center points. Using the rule of area dominance, the cells located on the border of each administrative region were organized into subordination levels [22,23]. ArcGIS 10.2 Zonal Statistics was used to create statistics for various land-use, spectral feature, epidemiological, and risk assessment data for each cell and in each time period. The data were saved accordingly in cell attribute tables, and the grid system for potential epidemic risk areas in the DTL region was generated from 2006 to 2016.

#### 2.2.2. Identification and Extraction of Snail Breeding Grounds

(1) Identification and extraction of environmental types of potential *O. hupensis* breeding grounds based on spectral features

This study mainly utilized environmental factors with significant sensitivity such as ground object types and regional environmental characteristics for key analysis. The vegetation index is a simple, effective, and empirical measure of surface vegetation based on spectral characteristics. Taking into account the feasibility and reliability of the vegetation index, we applied the research method of He, Zhang, and Tan, using normalized differential vegetation index (*NDVI*), green vegetation index (*GVI*), and brightness index (*BI*) in the analysis [28,29,30]. We processed the RS images to identify and analyze the environmental characteristics of the *O. hupensis* breeding grounds within the DTL epidemic area. The following equations were used in the calculations:

(1) $$NDVI=(TM_{4}-TM_{3})/(TM_{4}+TM_{3})$$

(2) $$GVI\;=-0.247TM_{1}-0.163TM_{2}-\;0.406TM_{3}+0.855TM_{4}\;+0.055TM_{5}-\;0.117TM_{7}$$

(3) $$BI=\frac{1}{3}{\left[(TM_{2}{)}^{2}+(TM_{3}{)}^{2}+(TM_{4}{)}^{2}\right]}^{\frac{1}{2}}$$

**Figure 2:**
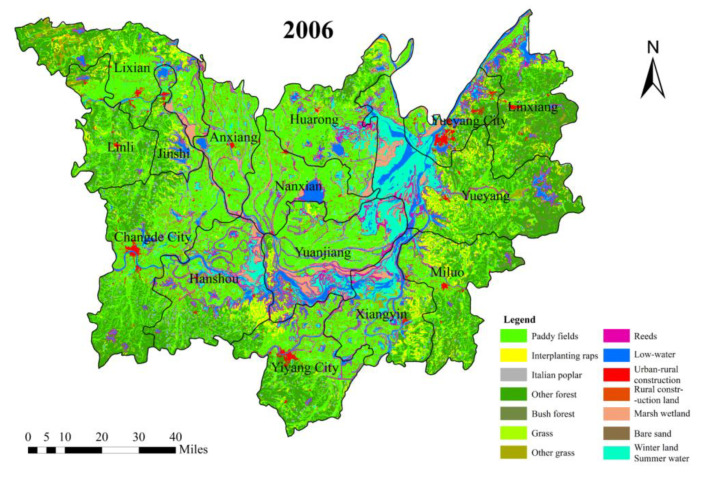

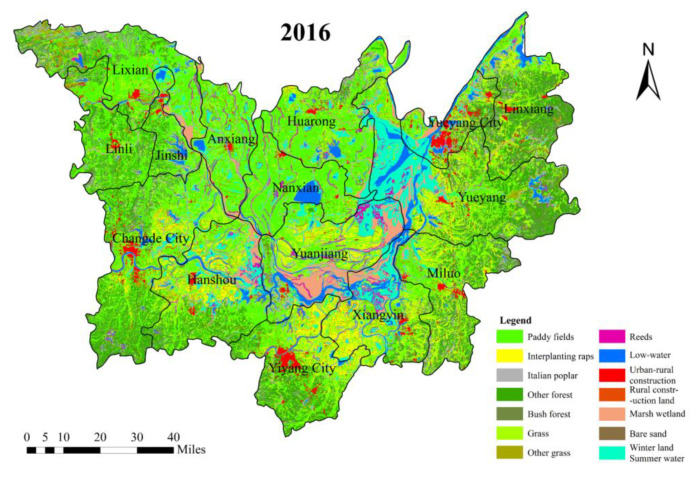
Distribution of environmental characteristics of the Dongting Lake (DTL) area from 2006 to 2016.

The environmental categories suitable for *O. hupensis* and schistosomiasis breeding were surveyed and classified. The main environmental types that could be suitable for the breeding of snails and schistosomiasis in the study area include paddy fields, reeds, Italian poplar forest, interplanting rape, and marsh wetland [29]. Based on the spectral distribution maps for *NDVI*, *BI*, and *GVI*, the spectral feature indexes of 900 samples were extracted. We then obtained the *NDVI*, *BI*, and *GVI* index averages for the nine environment types and their corresponding confidence intervals (95%) [31]. Based on the distribution characteristics of the three indexes, we conducted spectral index analysis and evaluated the land-use for the DTL area. Sample area verification and visual interpretation were used to improve the classification accuracy of the surface environment. We then identified and extracted the typical environment and secondary environment maps for the DTL area (2006–2016), as shown in Figure 2.

(2) Identification of potential *O. hupensis* breeding grounds in the DTL area from 2006 to 2016

In China, there is a lack of a unified identification standard for *O. hupensis* breeding grounds. Most studies detect *O. hupensis* breeding grounds using biological and specific environmental characteristics. For this study, we adopted concepts of relativity such as NDVI, BI, GVI, and snail density to predefine the snail breeding area. We collected the environment data from 2006 to 2016, recorded the extent (i.e., longitude and latitude coordinates) of the susceptible environment, and organized them into a susceptible environment checklist. The calculated average density of live snails in the susceptible area was 0.37/0.11 m^2^, and the variance was 0.29. The average value was used as cut-off; susceptible areas with density of live snails equal to or higher than the average value were considered suitable for the survival and reproduction of snails. The vegetation, soil, and humidity characteristics of these areas provide the necessary food, reproduction, and migration conditions for breeding snails. Therefore, we defined these areas as samples of breeding areas. Areas with snail density less than 0.37/0.11 m^2^ and greater than 0 possess basic conditions suitable for the survival of snails. These areas can become breeding grounds under certain circumstances and were designated as potential snail spreading areas. In the meantime, since the density of *O. hupensis* in other areas was 0/0.11 m^2^, we confined these places with low risk, which are not suitable for *O. hupensis*.

Through extracting the remote-sensing image of samples in the breeding area and diffusion area, we extracted and calculated their 95% confidence interval of the *NDVI*, *GVI*, and *BI* [32]. Threshold analysis found an overlap in the spectral index threshold of the breeding area and diffusion area. The overlapping area for vegetation indexes indicates that the susceptible environment corresponding to the overlap threshold of the *NDVI*, *GVI*, and *BI* was “the best location” for the breeding and spread of infectious sources (*O. hupensis* and Schistosoma). From an environmental perspective, these areas are most likely epidemic risk regions where schistosomiasis can occur or spread. Then, using the spectral data threshold distribution for the classification rule, potential epidemic risk areas were identified and classified into four categories: potential epidemic area, suspected breeding area, suspected spreading area, and other areas. Then, the samples of potential epidemic area, suspected breeding area, suspected spreading area, and the other areas were normalized according to the snail density data, and the breeding risk value of the sample area was obtained.

#### 2.2.3. Evaluation and Calculation of Potential Epidemic Risk of Schistosomiasis

(1) 2006–2016 epidemic index evaluation and calculation in potential risk areas

The epidemic risk assessment is a comprehensive evaluation based on the conditions of a particular area, reflecting the development trend of a given epidemic. This study synthesized the epidemiological data for 15 risk areas in the DTL using the epidemic index as a quantitative evaluation basis for schistosomiasis epidemic risk. From the 2006–2016 Annual Report of Schistosomiasis Control in DTL District, 13 parameters characterizing the environment, livestock conditions, and population demographics were selected for the index layer (e.g., snail inspection and the area of snails inside and outside the embankment, the area of susceptible regions inside and outside the embankment, the number and positive rate of blood and fecal tests, the number of farm cattle and the number of sick cattle, the number of patients). We aggregated indicators according to the calculating methods to control and eliminate schistosomiasis in China based on formulas with statistical significance. We used the MATLAB data processing software and EWM (entropy weight method) to calculate the formation entropy of comprehensive epidemiological indicators, weight coefficient, and the epidemic index for schistosomiasis epidemic risk in the 15 endemic areas of DTL from 2006 to 2016 [27,33,34]. Then, we extracted the evaluation index of the sample area, and used the above method to calculate and save the epidemic index.

(2) 2006–2016 susceptibility index calculation of potential risk areas

The significant differences in the distribution characteristics, patterns, and trends for schistosomiasis in different epidemic type areas are caused mainly by people–land conflicts resulting from interactions between potable and contaminated water. The land factor is the important carrier of epidemic trends, spreads, and human–land conflict. It reflects the situation that the land types, which exert positive and negative influence on schistosomiasis, combine and restrict others in specific areas. It is also an integrated expression of regional social and natural attributes. The functional nature of land determines the land cover type. Aside from being a major determinant of socio-economic activities, land/cover can significantly impact the survival of schistosomes and snails [35,36].

In this study, we introduced the concept of regional susceptibility risk. We used the spatial lag model (SLM) to establish an epidemic risk description method based on land-use types. We calculated the epidemic sensitivity coefficients *β_ij_*, which provide the relative estimate of the influence of different land-use types on the schistosomiasis epidemic in different regions. We calculated the susceptibility index *Y_i_* using the quantization formula:

(4) $$Y_{i}=\sum_{j=1}^{n}X_{ij}*\beta_{ij}+c_{ij}$$

where *c_ij_* is a constant term to quantify the comprehensive influence of various land types *j* in a specific area *i* in the development of a schistosomiasis epidemic. 

#### 2.2.4. Extraction and Storage of Data in Grid System

**Figure 3:**
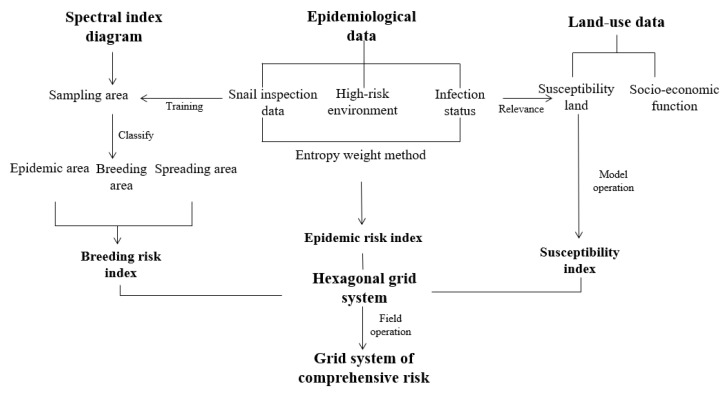
Research ideas and method frame diagram.

Based on the data of *O. hupensis* breeding risk and epidemic risk in the sample area, the distribution map of breeding risk and epidemic risk in the potential risk area with 30 m resolution was made by the kriging interpolation method. Then, the attribute fields in the mean value of breeding risk and epidemic risk from all grids are counted through the zonal statistical as a table in ArcGIS 10.2 software, as shown in Figure 3. In addition, we used the formula above-mentioned to conduct field operations on the land-use data that belonged to the grid system in the potential risk area in 2006–2016, and generated the susceptibility index for all hexagonal cells in potential risk areas from 2006 to 2016.The output results were saved as the grids of breeding risk, epidemic index, and susceptibility index attribute field for each area. More details on the calculation methods can be searched in the “Identification and risk monitoring of potential risk areas for schistosomiasis in DTL area” by Xu et al. in the Chinese Journal of Disease Control and Prevention [22].

#### 2.2.5. Quantification and Visualization of Comprehensive Risks in Epidemic Areas

The standardized *O. hupensis* breeding risk, standardized epidemic index, regional susceptibility index, and breeding risk were used as primary indicators, and their respective sub-indices were used as secondary indicators. We utilized Grey relational analysis (GRA) and the analytic hierarchy process (AHP) to obtain the weights of the primary and secondary indicators [37,38,39] and combined the discretized grid data with three types of risk weights to get the comprehensive risk value $G_{i}$. We then established a spatial rectangular coordinate system, where $x_{i}$, $y_{i}$, and $z_{i}$ indicate the epidemic risk, breeding risk, and the weighted quantitative value for susceptibility risk, respectively. We calculated the Euclidean distance [40,41] from all grid coordinate points to the central reference point ($x_{\max}$, $y_{\max}$, $z_{\max}$) and used the P-quantile division method to divide the value of the corresponding point in each grid into five grade intervals. Based on the classification requirements from the Law of the People’s Republic of China on Response to Emergencies, the areas were subdivided into five levels based on their comprehensive risk values: Level I (extremely high-risk area; red), Level II (high-risk area; orange), Level III (moderate-risk area; yellow), Level IV (low-risk area; blue), and Level V (risk-free area; white). Finally, the comprehensive risk assessment and early warning map for schistosomiasis in the DTL was generated.

### 2.3. Data Source and Extraction of the Potential Risk Study Area

Based on the definition of potential risk area, the research data should contain the maximum range of the intersection of these areas, which include 15 schistosomiasis endemic cities (also counties and districts), the “winter land, summer water” area where *O. hupensis* thrive, and the contradiction area between potable and infected water. We obtained the annual schistosomiasis endemic data for 15 schistosomiasis endemic cities (also counties, districts) in the DTL area from the Hunan Province Schistosomiasis Control Center and acquired the land-use and land-cover change (LUCC) data from the Resource and Environment Science and Data Center of the Chinese Academy of Sciences. We downloaded Landsat5 and Landsat8 remote sensing images during the low-water and high-water periods in 2006–2016 from the geospatial data cloud at 123-40, 123-39, 124-39, and 124-40 orbit and with 30 m pixel size. As Landsat5-TM images and Landsat8-OLI images were used for 2006–2010 and 2010–2016, respectively, the images had to be preprocessed to ensure the mutual unity of the data. Analyzing the historical water level data of the DTL from 2006 to 2016, we found that the highest water level was at 34.47 m, which occurred in July 2016, while the lowest was at 20.38 m in December 2015. 

**Figure 4:**
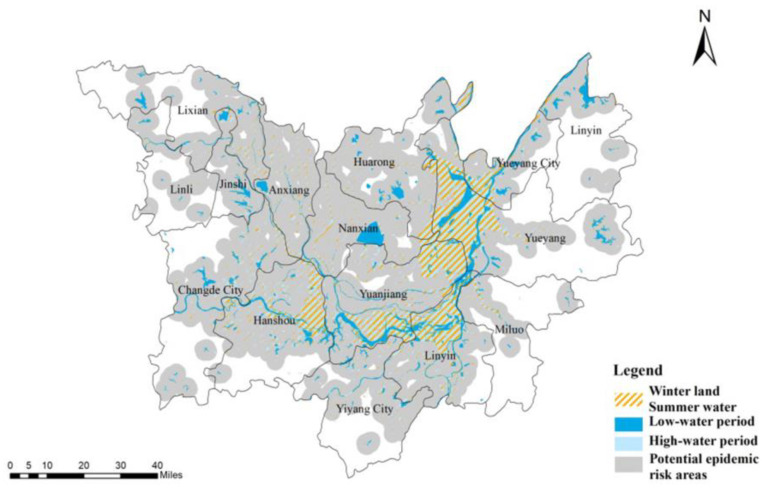
Regional map of potential risk of schistosomiasis in the DTL area from 2006 to 2016.

Modified normalized difference water index (MNDWI) was used to enhance the water body effect and extract the water area from the RS images at that time, which were then converted into polygons in ArcGIS 10.2. Using overlay analysis and RS image processing, the largest extent of the “winter land, summer water” area for 2006–2016 was acquired [40,41]. We demarcated this designated area with a 4-km buffer zone (diameter of daily activities of the residents, livestock, and poultry in the lake area, obtained through the questionnaire). Moreover, we amended the buffer zone using several kinds of data to build classification rules. These data include the AsterGDEM data with 30 m resolution ratio in DTL area, EVI index, and the distance from the water [42]. The spatial data were superimposed and analyzed in ArcGIS 10.2, and the potential epidemic risk areas in DTL were determined, as shown in Figure 4.

## 3. Results

### 3.1. Spatial Distribution Analysis of Potential O. hupensis Breeding Grounds

**Figure 5:**
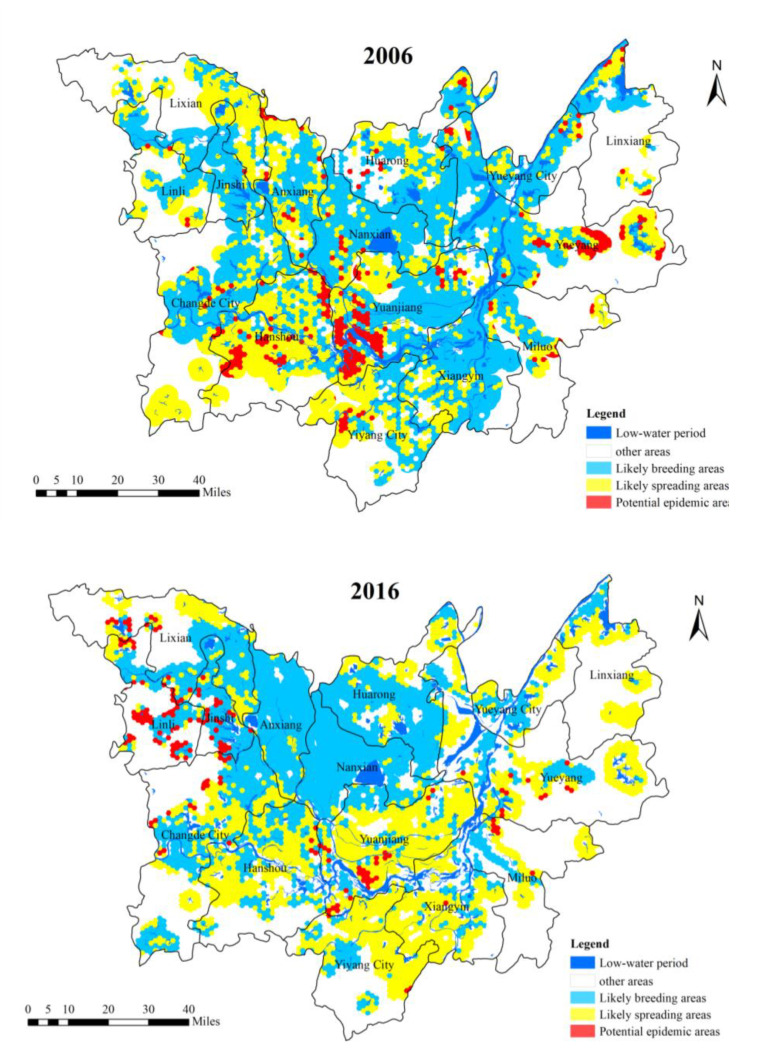

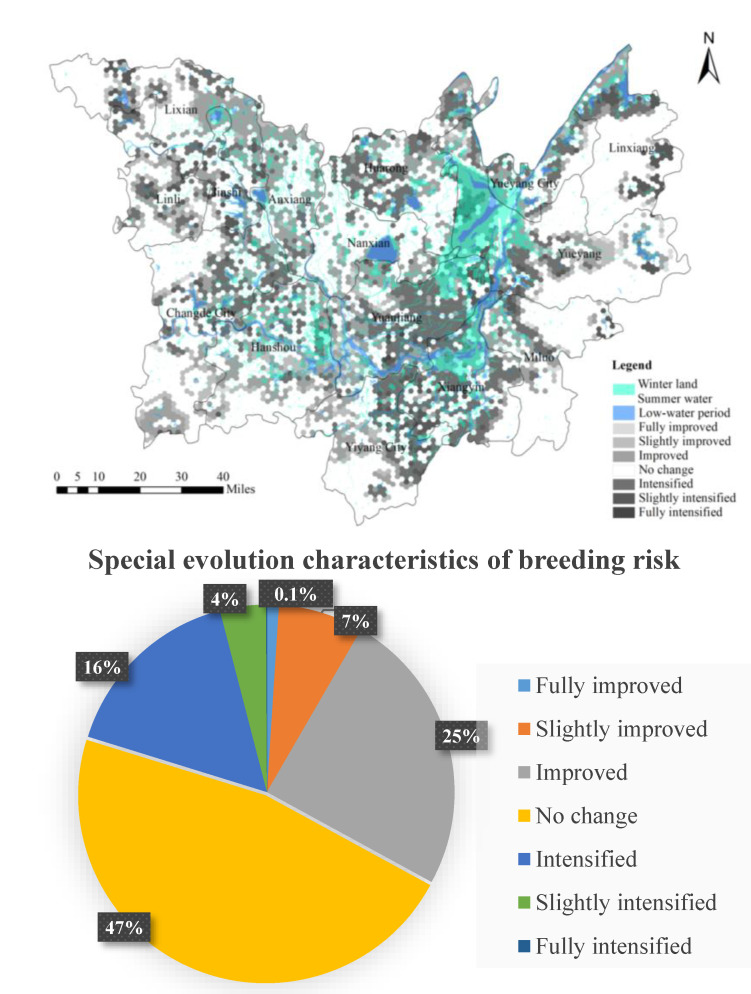
The spatial evolution characteristics of potential *O. hupensis* breeding grounds in the DTL area from 2006 to 2016.

Using the generated map for potential *O. hupensis* breeding grounds and the calculation results (as shown in Figure 5), we assessed the overall distribution pattern and dynamic changes of potential epidemic areas, likely breeding area, likely spreading area, and other areas. For 2006–2016, the four area types exhibited varying spatial evolution characteristics. In the areas of evolution, improved area with decreasing breeding risk was in the majority, which accounted for 33%. The area with increasing risk accounted for around 20%. In both of these areas, the changes were minor, either slightly improved or slightly intensified. In terms of the spatial characteristics of risk evolution, the breeding risk generally exhibited a declining NE to SW pattern: high in the eastern and northern regions and low in the southwestern portion. The areas with increased breeding risks were mostly concentrated along the east DTL, particularly in Yiyang City, Yuanjiang City, Yueyang City, Xiangyin County, and Yueyang County. There were also a number of scattered distributions in Jinshi City, Linli City, and in the northern part of Changde City, located in the northwest DTL. 

The four area types shifted from fragmented distributions into more centralized configurations. For example, suspected spreading and suspected breeding areas transformed from an interlaced, scattered, and mixed distribution in 2006 to a north–south pattern in 2016, indicating the evolution of centralized contiguous pieces had basically formed. The other scattered areas become homogenization continuously by the surroundings. The potential epidemic focus areas evolved from multiple patches and scattered points in 2006 to compact and denser clusters in the central region of the DTL, particularly in Yuanjiang City, Jin City, and Li County. A large area around east DTL, which includes large portions of “winter land, summer water” areas, changed from being a suspected spreading area in 2006 to a suspected breeding area in 2016. This shift suggests that due to some natural or anthropogenic factors in the lakeside and waterfront areas, more land became suitable for *O. hupensis* breeding. This may also mean that the snail population exhibits lakeside and hydrophilic agglomeration.

Despite the potential epidemic focus areas becoming more clustered, their aggregate size declined. To some extent, this clustering and shrinkage of potential epidemic focus areas suggest that the spatial distribution of snail density has distinct regional differentiation. This also implies the weakening of the spatial mobility of the snail population, the growing independence of solitary groups, and the increasing dependence of the snail population to the local environment. Such spatial characteristics are more conducive for *O. hupensis* detection and extermination, which can help schistosomiasis control staff more efficiently minimize snail population and reduce potential epidemic breeding areas [43,44].

### 3.2. Analysis of Comprehensive Risk Evolution Characteristics of Schistosomiasis Epidemic 

**Figure 6:**
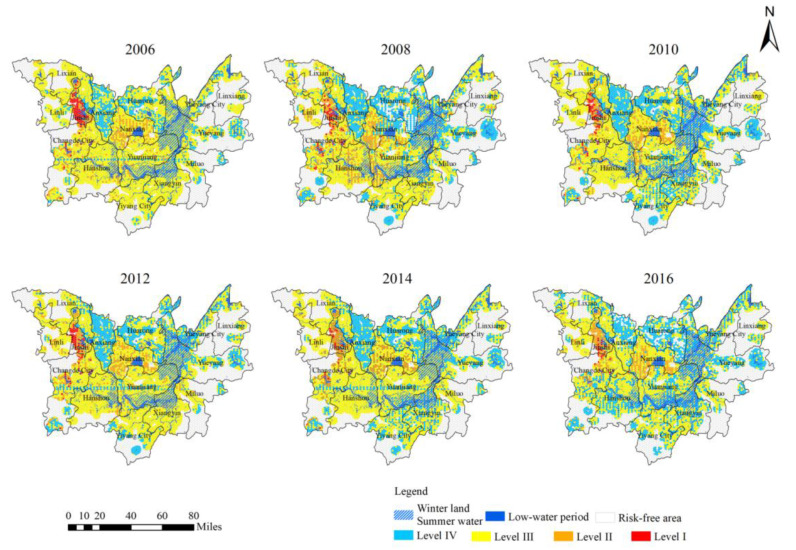
Spatio-temporal pattern of schistosomiasis comprehensive risk evolution in DTL from 2006 to 2016.

As shown in Figure 6, the spatial distribution of schistosomiasis risk in the DTL area can be characterized as having an overall pattern of being high in the core area, low in the peripheral area, high in the periphery of large lakes, low in other areas, high in the west Dongting area (northwest of the central part), and low in the east Dongting area (northeast of the central part). Level I and Level II risk areas were distributed in the following regions: the area around the Maoli Lake and West Lake in Jinshi City, which lies in the west DTL area; area north of the Liuye Lake in Changde City; and the area around Datong Lake in Nanxian County in the hinterland of DTL. The low-lying embankment around the Lishui Basin was also included. The Level III and Level IV risk areas were mainly distributed in south and northeast DTL, which includes Yueyang City, Yiyang City, Huarong County, Yuanjiang City, Xiangyin County, Yueyang County, and Anxiang County. The spatial interaction between Level III and Level IV areas increased considerably, with recurring conversion between risk types. The overall pattern for the Level III risk area spread from southwest DTL to the northeast, which gradually shifted into a fragmented reduction. The Level IV risk area exhibited a progressive and fluctuating increase in size, gradually extending to neighboring areas.

We utilized local spatial autocorrelation analysis (Getis-Ord Gi*) in the GIS exploratory spatial data analysis method (ESDA) to characterize the spatial autocorrelation of epidemic risk in potential risk areas. We adopted the Z (Gi*) value to identify the spatial evolution pattern of high-high agglomeration (hot-spots) and low-low agglomeration (cold-spots). As shown in Figure 6, the epidemic area was divided into seven types based on the positive and negative significance of Z (Gi*): Hot-spots 90%, Hot-spots 95%, Hot-spots 99%, Cold-Spots 90%, Cold-Spots 95%, Cold-Spots 99%, and no spatial correlation. 

**Figure 7:**
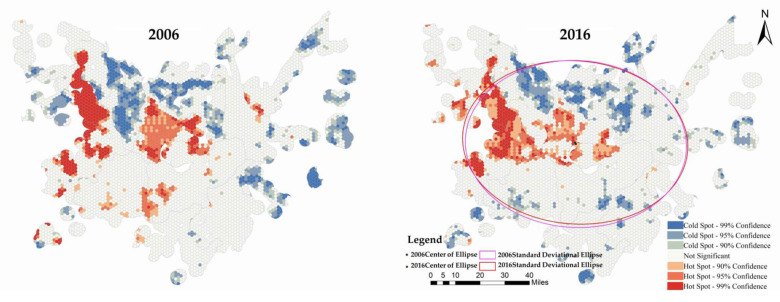
Getis-Ord Gi* and standard deviational ellipse of comprehensive risk in epidemic areas from 2006 to 2016.

The high- and low-value distributions of epidemic risk in potential risk areas exhibited distinct spatial correlation. As shown in Figure 7, the grid count for hot-spots and cold-spots in 2006 were 821 and 1123, respectively, accounting for 12.57% and 17.19%. In 2016, the grid count was 824 for hot-spots (12.61%) and 954 for cold-spots (14.60%). Compared to 2006, hot-spot areas with 99% significance decreased by 22.64%, with 95% significance increased by 0.37%, and with 90% significance increased by 57.59%. For cold-spot regions, areas with 99% significance decreased by 18.73%, with 95% significance decreased by 18.92%, and with 90% decreased by 4.9%. The risk agglomerations exhibited a gradual weakening trend. 

The distribution and evolution of high- and low-risk values in potential risk areas showed noticeable spatio-temporal continuity. In 2006, the cold-spots were mainly distributed in the north and east DTL, while the hot-spots were in the central and west DTL and along the lake. In 2016, the cold-spots were mainly in the north and south peripheral areas of the DTL, while the hot-spots were along the west DTL. The cold-spot region can be characterized as having Huarong County and Anxiang County as the core and a scattered distribution in other peripheral areas. From 2006 to 2016, while the extent of the core cold-spot region declined, and the patches along the margins dropped, the number of cold-spot patches grew significantly. This means that the spatio-temporal evolution pattern for low risk areas shifted from a huge decline in concentrated and contiguous areas to distinct breakthroughs in a number of areas. The hot-spot areas had a concentrated distribution centered in Jinshi City, Nanxian County, the southern part of Huarong County, and the northern area of Changde City. The coverage area of the core hot-spot area declined, while the degree of fragmentation increased. The apparent reduction of scattered patches in the periphery indicates that the spatio-temporal pattern of high risk areas shifted from concentrated contiguous regions into more fragmented and shrinking areas, with increasing fragmentation reduction of the interior core region. 

The standard deviational ellipse method was used to present and analyze the comprehensive risk distribution and evolution direction of potential risk areas. The standard deviational ellipse involves key elements such as the position of the center point, the semi-major and semi-minor axes, and the azimuth angle. The position of the center point represents the center position of the comprehensive risk element in the epidemic area. The semi-major axis indicates the discrete degree of the data distribution in the principal direction, while the semi-minor axis represents the discrete degree of the data distribution in the sub-principal direction [45,46]. The larger the difference between the semi-major and semi-minor axes (the greater the flattening), the more distinct the directivity of the data. Conversely, the closer the values of the semi-major and semi-minor axes, the less obvious the directivity. As shown in Figure 7 and Table 1, the difference in ellipse coverage area and in ellipse direction angle between 2006 and 2016 were small. From 2006, the coverage area in 2016 decreased by 0.36%, and the direction angle shifted by 1.65°. The geographic coordinates of the center of the ellipse moved from (29.174° N 112.418° E) to (29.183° N 112.415° E), and the overall offset shifted from west to north. The semi-major axis increased by 1.429 km while the semi-minor axis decreased by 1.252 km, indicating increased flattening of the ellipse. The risk distribution and the evolution of potential risk areas were relatively stable, with the center shifting northwest and the distribution axis extending from the northeast to the southwest. The evolution was initially along the east–west direction, and then shifted to a north–south trend. The spatial risk distribution featured enhanced concentricity along the major axis and increased dispersion along the minor axis.

### 3.3. Comprehensive Risk Classification Criteria of Schistosomiasis in DTL Area

As shown in Table 2, we used the comprehensive risk value quantification and grading rules in Section 2.2.5 to evaluate all grids in the study area and statistically analyze the different attribute data in each grid. We analyzed the characteristics of extremely high-risk (Level I), high-risk (Level II), and moderate-risk (Level III) regions.

In terms of epidemiological data, the extremely high-risk (Level I) areas had comparatively more infected people and animals, large susceptible environments, and considerable snail populations, conducive for accelerating the occurrence and spread of schistosomiasis. Highly susceptible land-use types accounted for a relatively large proportion of Level 1 areas, making these places highly conducive to the occurrence and spread of the epidemic [43]. In terms of land-use, Level 1 areas were dominated by waterfront planting areas, waterfront marsh wetlands, farmland cultivation areas, and agro-forestry areas. These areas provide ecological functions such as hydrological regulation, agricultural production, and ecological conservation.

According to the epidemiological data, the risk index for human and animal infection, the proportion of susceptible environment, and the quantity of oncomelonia snails inside and outside the embankment were possibly higher than the global average, which could significantly affect the spread and development of epidemics. A large proportion of high risk (Level II) areas were highly susceptible areas including cultivated farmlands, waterfront planting areas, agro-forests, and other socio-economic functional areas. Much of the Level II areas were used mainly for agricultural production, and a small portion is reserved for ecological conservation. 

Through the sampling survey, the epidemic index and susceptibility index of moderate risk (Level III) level areas were found to be near the global exponential average in DTL areas. The number of human and animal infection, the proportion of susceptible environment, and the number of snails inside or outside the embankment were at a medium level, while snail density fluctuated slightly around 0.37/0.11 m^2^. These areas could play an intermediary role in the prevalence and development of the epidemic. When the land-use in these regions was extracted and analyzed, we found that the land-use types with higher sensitivity index β accounted for a moderate proportion, which could promote the occurrence and transmission of the epidemic. Level III areas were mainly cultivated farmlands, mixed cultivated and residential areas, and waterfront planting areas. Additionally, Level III areas were supplemented with mixed agricultural and forest lands, flood plains, and other ecological areas, mainly used for agricultural production, water, and soil conservation, and some non-agricultural functions.

## 4. Discussion

### 4.1. Regional Characteristics and Control Strategies for Medium- and High-Risk Areas

(1) Level I risk areas

The major environments in Level 1 areas included farmlands, marsh wetlands, “winter land, summer water” environment, grass islands, shrub forests, and sparse woodland environments. In terms of distribution, the extremely high-risk epidemic areas were mainly situated close to Lake Basin, within 2–6 km from concentrated settlements. The area falls somewhere in between usually active regions for humans and animals and low-activity areas. The interaction between potable and contaminated water for Level 1 areas was relatively low. Human and livestock activities in the area were all non-daily ones rather than normal production and life. Level 1 areas have not been subjected to anthropogenic modifications. It is difficult for schistosomiasis control work to cover this area and is easily ignored by the objects that conduct activities in it [47,48]. These are highly critical areas that need to be prioritized and closely monitored. 

Combined with vegetation cover and epidemic index characteristics, we believe that the key to reducing the comprehensive risk is to prevent snail breeding. The better methods include optimizing the planting structure, adjusting the vegetation index, and enriching the means of snail destruction. Therefore, the prevention and control model for Level 1 areas should include flood prevention, waterlogging mitigation, and water and soil conservation measures. The control strategies should consist of developing the beach economy, afforestation, and snail control, and should be supplemented by measures such as fencing and sealing off the island, trenching and draining, and engineering snail control. The policies should adhere to principles of protection-oriented, moderate development, and unified management and control for ecologically sensitive areas. Governance and management for Level 1 areas should focus on water conservancy, schistosomiasis control, forest maintenance, and environmental protection to ensure ecological and biodiversity conservation. An early warning and monitoring system is particularly important for high-risk areas. The government should invest more in personnel development, facility upgrading, and technological modernization to help monitor the local snail population, identify risk factors, and promptly respond to the epidemic spread [43,44].

(2) Level II risk areas

In terms of environmental types, Level II risk areas were dominated by farmland, “winter land, summer water”, and grassland environments and supplemented by shrub forest and sparse woodland environments. These lands were mainly distributed in West DTL, Nanxian County, and Yuanjiang River and included low potable and contaminated water interaction areas. They tended to be near lake basins, within 2–6 km distance from concentrated settlements or urban-rural construction land boundaries, the area falls somewhere between the usually active regions for humans and animals and low-activity areas. Although the high-risk areas had similar distribution characteristics to extremely high-risk areas, they were more widely distributed and had faster spatial evolution rates, which are very challenging in epidemic monitoring and risk management.

It is rational to use the adaptability of agricultural production factors in waterfront areas and the incentive mechanisms of economic development in the epidemic area. The regional agriculture, forestry, and animal husbandry structures must be improved; embankments should be developed and utilized; and the swamp and grassland environments must be modified to reduce ideal *O. hupensis* breeding grounds and prevent the build-up of contaminated water in the embankment. The management of forestry areas should pay attention to the use of forest space. It is necessary to choose vegetation with the function of killing and driving out *O. hupensis* to carry out under-forest interplanting. This action can form a system with trenching waterlogging, lifting continents, and lowering beach engineering. At the same time, through the establishment of wetland parks and natural ecological reserves, wetland resources and grassland environments are protected to guide people to enter in an orderly and safe manner and reduce the occurrence of sudden and random epidemics.

(3) Level III risk areas

The environments found in Level III areas included farmland, grass island, reed, shrub forest, and sparse woodland environments. These lands were mainly concentrated in the west and south DTL including urban and rural construction lands, concentrated rural settlements, and surrounding vicinity within 6 km, and are commonly used by residents, livestock, and poultry. This covers the scope of high- and low-activity areas for residents, livestock, and poultry. The comprehensive risk in this area has a fast change rate. It also has frequent expansion and contraction and covers the area of potable and contaminated water interaction intensity at all levels. Frequent human and livestock activities occur in these areas including production and other daily-life activities, and many environment types can be found in this risk group.

The large area indicated by spectral characteristics, a large number of land-use types with high epidemic density, and the complex social and economic activities of residents bring up many uncertainty elements. All these elements caused the prevention and control of the environment in risk areas, or *O. hupensis* breeding areas were more difficult and less efficient [49]. Residents in Level III areas have to be guided on which areas are safe. The government should consider introducing policies such as relocation subsidies and increased personnel training for agricultural professionals. Measures should be taken to speed up land transfer and management and develop collective agriculture and large-scale contract agriculture. Changes in the embankment area should be introduced including modifying the area’s backward single-reed and fishing economy, expanding the pilot scope for shrimp, crab, fish, waterfowl, and fungus breeding, developing economic crop cultivation, promoting mechanization and industrialization, and using engineered intervention to control snail population and make the area less conducive to snail breeding and habitation [26].

### 4.2. Driving Factors of Comprehensive Risk 

The driving factors of the comprehensive risks of schistosomiasis epidemic involve natural factors such as temperature, humidity, soil properties, light intensity, vegetation types, and vegetation coverage. They are also closely related to human factors such as regional economic development, population health, mode of production and lifestyle, prevention awareness, and governance. Previous studies such as Kristensen [50], Tayo [51], and Liu et al. [52], extracted ecological factors (e.g., vegetation, climate change, and precipitation) and combined them with local epidemiological data to determine the regional distribution of schistosomiasis and identify environmental risks. 

A schistosomiasis epidemic originates from the snails’ breeding environment. Breeding environment refers to the macro-ecosystem and micro-ecological environment suitable for the survival, development, and reproduction of snails and schistosoma. It shows the interaction between schistosoma eggs, miracidia, cysts, cercaria, adults, and the natural environment, and it serves as the natural carrier of schistosomiasis infection in humans and animals. Aside from the breeding environment, comprehensive risks are also affected by human factors and land factors. Yingnan Niu et al. found that there were essential correlations between land use, ditch density, production methods, and the infection rate of residents [45,46]. Human factors are the driving mechanisms for the spread of schistosomiasis. Particular socio-economic economic activities may cause significant spatio-temporal changes to schistosomiasis risks. The human-to-land conflict between potable and contaminated water causes spatial interaction between snail breeding grounds (or contaminated water) and human activities. 

Schistosomiasis is a highly social disease. Human factors affecting the spatio-temporal aspects of this disease include regional socio-economic level, sanitation, prevention and control awareness, the population’s production mode and lifestyle, and the intensity of epidemic prevention and control. Jun Yang used a questionnaire survey to evaluate the acceptability and effect of afforestation and improving farmers’ lifestyles as *O. hupensis* reduction measures. As the main carrier for the development and spread of the epidemic, land is a comprehensive expression of a region’s social and natural attributes and can significantly inhibit or promote the prevalence and breeding of the epidemic. The functional nature of the land affects the type of cover on its surface, which can positively or negatively impact snail and schistosome populations [53].

Using simulation analysis of *O. hupensis* breeding grounds and the spatio-temporal evolution of epidemic risks, the epidemiological characteristics, distribution pattern, socio-economic, and natural parameters of risk areas can be more accurately evaluated at different levels and be used in developing epidemic prevention and control strategies.

This study has several limitations. *O. hupensis* breeding and migration are highly complex processes with many changeable variables. Much can still be improved in the accuracy of detecting *O. hupensis* and identifying breeding areas. In addition, the definition of potential epidemic risk remains an open debate. The selection of factors to be evaluated is limited by the statistical caliber in the epidemic region and external parameters. At the same time, studies on the universality of social-economic and natural parameters in specific regions need to be strengthened.

## 5. Conclusions

This study evaluated potential schistosomiasis risk areas in Dongting Lake and explored the factors affecting the occurrence and transmission of the disease. Using remote sensing and geospatial analyses, this study explored the “locational preference” and breeding patterns of *O. hupensis* and how they relate to epidemic risks. Data on snail population, vegetation, environmental attributes, water conditions, and soil characteristics were integrated to identify suspected *O. hupensis* breeding areas and epidemic susceptible zones at the macro-level using quantitative analysis. This study helps improve the sensitivity and evaluation accuracy of risk identification, particularly in low-level epidemic areas. The main conclusions of the study are as follows:

(1) From 2006 to 2016, the spatial change of potential *O. hupensis* breeding grounds showed a weakening trend from the eastern and northern areas of DTL to the southwestern area. In the four types of risk areas, most of the improved areas exhibited a decrease in risk over time. For those that exhibited some change in breeding, the changes were mostly minor. More potential *O. hupensis* breeding areas emerged in east DTL, exhibiting some lakeside and hydrophilic agglomeration characteristics. The snail breeding areas evolved from fragmented to centralized distribution and had distinct regional (spatial) differentiation. The results also indicate the weakening of the snail population’s spatial mobility, the increasing independence of single snail groups, and the growing dependence of snail populations on their local environment.
(2) The spatial risk distribution in potential risk areas in DTL exhibited an overall pattern of high in the core area, low in the peripheral area, high in the periphery of large lakes, low in other areas, high in the west Dongting area, and low in the east Dongting area. The cold-spot areas had Huarong County and Anxiang County as the core, with scattered distributions in peripheral areas. From 2006 to 2016, the core cold-spot region declined, the marginal cold-spot patches shrank, but the number of patches significantly increased. The risk distribution’s center shifted to the northwest, and the distribution axis extended from northeast to southwest. The evolution was initially in the east–west direction and then shifted to the north–south direction. The spatial risk distribution exhibited enhanced concentricity along the major axis and increased dispersion along the minor axis.
(3) Using the epidemiological, socio-economic, and environmental characteristics of extreme high-risk, high-risk, and modern risk areas, we put forward targeted and differentiated strategies to prevent and control the occurrence and spread of the schistosomiasis epidemic. These strategies and measures can reduce the *O. hupensis* brewing risk, aesthetic risk, and susceptibility of land use in various regions. They can also be used to promote socio-economic development and environmental protection in different regions.

## Figures and Tables

**Table 1:** Main parameters of comprehensive risk standard deviation ellipse in potential epidemic risk areas.

Attributes	2006	2016
Shape_Length	399.203 km	400.390 km
Shape_Area	12,314.978 km^2^	12,270.277 km^2^
Center X	112.418° E	112.415° E
Center Y	29.174° N	29.183° N
XStdDist	72.042 km	73.471 km
YStdDist	54.415 km	53.163 km
Rotation	92.817°	94.468°
Oblateness	0.245	0.276

**Table 2:** Characteristics of the comprehensive risk areas of schistosomiasis in the DTL area.

Comprehensive Risk Level	Comprehensive Risk Value	Environmental Spectral Characteristics	Epidemic Index Characteristics	Susceptibility Index Characteristics
Level I (Extremely high-risk area)	8.92–12.09	BI: 29.31–39.45 GVI: 11.69–42.51 NDVI: 0.15–0.35	≥0.44	≥3.92
Level II (High-risk area)	6.10–8.81	BI: 27.30–44.64 GVI: 3.45–59.37 NDVI: 0.09–0.40	≥0.35	≥1.5
Level III (Moderate-risk area)	4.68–6.05	BI: 18.46–29.31 ∪ 39.45–46.36 GVI: −19.27–11.69 ∪ 42.51–62.55 NDVI: −0.14–0.15 ∪ 0.35–0.61	0.07–0.46	0–2.5
Level IV (Low-risk area)	2.63–4.64	BI: 18.46–27.30 ∪ 42.69–46.36 GVI: −19.27–15.68 ∪ 59.51–62.55 NDVI: −0.04–0.09 ∪ 0.39–0.61	≤0.35	≤1.5

## Data Availability

The data presented in this study are available on request from the corresponding author. The data are not publicly available as the data also forms part of an ongoing study.
